# Synesthesia and Migraine: Case Report

**DOI:** 10.1186/1471-2377-10-121

**Published:** 2010-12-07

**Authors:** Karl B Alstadhaug, Espen Benjaminsen

**Affiliations:** 1 Department of Neurology, Nordlandssykehuset, Bodø, Norway; 2 Institute of Clinical Medicine, University of Tromsø, Tromsø, Norway

## Abstract

**Background:**

Synesthesia is, as visual migraine aura, a common and fascinating perceptual phenomenon. Here we present a unique case with synesthesias exclusively during visual migraine auras.

**Case presentation:**

A 40-year-old woman with a cyclic mood disorder had suffered from migraine with visual aura for several years. On several occasions she had experienced "mixing of senses" during the aura phase. Staring at strong bright light she could experience intense taste of lemon with flow from the salivary glands.

**Conclusion:**

Acquired synesthesia, exclusively coincident with migraine aura, gives support to the idea of an anomalous cortical processing underlying the phenomenon.

## Background

Synesthesia, the phenomenon of impulses affecting one sense modality, giving a sensation that is normally experienced by stimulation of another sense modality, affects at least one per 100 [1]. It was described in Nature in 1880 [2], and more than a hundred years later, experimental studies validate it as a genuine perceptual experience, and not merely a metaphoric description or a memory association [3-5]. The neurophysiological mechanisms underlying synesthesias are largely unknown, but there are two main theories: cross-activation of the relevant perceptual areas due to a) cross-wirings that were not disconnected after birth or b) anomalous processing in perceptual association areas [5,6]. Positron emission tomography studies [3] and functional magnetic resonance studies [4] performed on patients who report seeing colors when exposed to auditory stimuli have shown activation in several visual-associated cortical areas. Here we present a unique case with synesthesias exclusively during visual migraine auras.

## Case presentation

A 40-year-old graphic designer with a bipolar disorder type II had suffered from regular attacks of migraine with and without visual aura over the last six years. Her mother suffered from typical visual aura without headache. Attack treatment with naproxen, rizatriptan and zolmitriptan had been unsuccessful. The efficacy of prophylaxis with propranolol was also questionable. Two years prior to the neurological consultation, the patient's psychiatric problems had piled up, and by that time she suffered two migraine attacks per week. She had been given increasing doses of a combination of codeine and paracetamol, but no pain relief was achieved. A genetic test showed that she was a poor metabolizer (CYP2D6, 2549A > del). Later the same year she received her psychiatric diagnosis. Mood-stabilizing treatment with valproate 500 mg/d was initiated. According to the patient, this halved the migraine attack frequency, and by the time of her consultation she experienced one attack per week in average. She now used petidine (100 mg) against the migraine headache.

The patient was eloquent and described typical migraine, but the aura was curious. She experienced what she characterized as "surrealistic phenomena" never lasting less than two and never more than 30 minutes. This was almost always followed by migraine headache within one hour. On several occasions she had experienced what she called "mixing of senses". A flickering with some sparkling was noticed at the rim of the upper lateral visual field(s). There was no classical spread of the visual disturbance and it never left a scotoma. However, during it she could experience a short lasting grayish central scotoma triggered by loud and high pitch sounds, such as the cry of children. This could make reading difficult. She was unable to tell how long the scotoma could last and nor could she give account for the temporal relationship to the scintillations. More conspicuous was an intense taste of lemon with flow from the salivary glands when she stared at strong bright light, especially light rods in the ceiling. This was also short lasting but could occur several times throughout the aura, and as she stated: "the flickering is present all along and seems to come from within", but "the other is caused by external stimuli and may be avoided". The aura was sometimes also associated with a short-lasting moody or euphoric feeling. She had no other types of synesthesias, and experienced no other illusions, hallucinations or psychotic symptoms. Neurological examination, cerebral MRI, standard and sleep-deprived EEG were all normal.

## Discussion

Similar to several other reports of patients with synesthesia, the present patient had a creative job [7]. She also had a psychiatric diagnosis. Can we be sure that the patient reported genuine synesthesia and not an acquired memory association? In fact, taste as the concurrent perception of vision in synesthesia is considered rare [8], and that colors may have a profound effect on the perceptions of odors (and taste) is well-known, and the association between yellow and lemon seems to be robust [9]. It is well known that bipolar disorders and migraine are associated [10]. Morrison found that seven of 46 female patients who were referred due to migraine experienced abnormal perceptions (smell and/or taste hallucinations and distorted body image). Six of the seven had an actual or previous affective disorder [11]. All of them experienced either depression or euphoria during their attacks. In another study, six out of 12 patients with bipolar disorder suffered from hallucinations of smell [12]. This was interpreted as psychomotor epileptic symptoms. In the present case epilepsy was considered, but found unlikely. Usually, synesthesia appears to be present across the individual's whole lifetime, but it is well known that the use of hallucinogenic substances may cause enhanced perceptions and induce synesthesias [13]. There is no reason to believe that this was the case in the reported patient. She used petidine only after headaches developed. We believe that present case is the most consistent description of genuine acquired synesthesia associated to migraine aura so far reported. Sacks described two migraineurs with synesthesia, one who experienced a single episode compatible with "a synaesthetic equivalence between auditory stimuli and visual images" [14], and another (a painter) who experienced synesthesia independent of the aura phase [15]. Podoll and Robinson reported about a female art teacher who experienced auditory-visual synesthesia [16]. Years apart, on three separate occasions, she had been woken up by the alarm clock experiencing migraine headache and a pulsating "coloured optical pattern" in the central field of her vision that was synchronized to the sound. A sense of impaired central vision is not uncommon during intense headache, but in our patient both the auditory-visual and the visual-gustatory stimuli mixture occurred prior to the headache. She experienced basic perceptions typical for synesthesia, and not vivacious hallucinations. Valproate had a beneficial effect on both the affective disorder and migraine [17], but it did not remove her abnormal perceptions. Recently, a female patient with synesthesia and a cyclic mood disorder was reported in the British Medical Journal [18]. Her synesthesia was initially interpreted as a psychiatric symptom. That case illustrates the danger of interpreting a common and harmless perception as a psychotic phenomenon [19]. Unfortunately the information constituting the basis for the present patient's psychiatric diagnosis is not available.

To explain how synesthesia could be related to migraine in our patient is challenging. Since it occurred exclusively during visual aura it is plausible that cortical spreading depression (CSD) may have caused it. Based on our current understanding of CSD (and aura), with its bimodal (positive symptoms followed by negative symptoms) and spreading characteristics [20], it seems unlikely that the synesthesias may have been aura symptoms per se. Besides, the migraine aura is apparently independent of external stimuli in contrast to synesthesia which depends on it. It is, however, possible to imagine that CSD temporarily may alter networks involved in cross-modal perception, making the brain susceptible for synesthetic phenomena. Whether an underlying congenital neuronal hardware is required for this to happen is unknown. Actually, visual evoked potentials are elicited in human neonates given auditory stimuli, and pathways between auditory and visual brain centers have been documented in neuroanatomical studies of young kittens [21], indicating that a congenital neuronal hardware for at least auditory-visual synesthesia may exist. In the present case it seems more unlikely since she experienced two types of synesthesias, and both were acquired. The audio induced central scotoma may reflect a synesthetic "release phenomenon", alternatively only increased attention to a classical negative visual aura symptom. Even though the patient's scintillations did not evolve with a gradual spread which is typical for CSD, this may have occurred unnoticed until the positive hallucination grew big enough in the lateral eye field. The taste of lemon in our patient was clearly a positive phenomenon and more difficult to explain. The cross-modal correspondences between vision and taste have not been well described, but the human taste region is probably located in the insulo-opercular region [22]. An alternative viewpoint to a congenital miswiring underlying the synesthesias, is that CSD temporarily causes an anomalous processing in normal perceptual association areas. All of the patient's reported complex internal sensations were actually linked to the temporal lobe [23], and it is tempting to suggest that cortical spreading depression crossing the occipital-temporal junction, an area important for integration of both vision, taste and sound, one way or another indirectly may have induced synesthesias. Based on the prevalence in the general population, about 2 in 1000 migraineurs would experience some sort of synesthesia, most of them of the visual type. Further reports and research on this subject are required.

## Conclusion

The present case report adds a little piece to a great puzzle. Acquired synesthesia coincident with migraine, appearing only during visual aura (and thus cortical spreading depression), gives more support to the idea of an anomalous cortical processing than to a congenital miswiring underlying the phenomenon.

## Consent

Written informed consent was obtained from the patient, and a copy of this is available to the Editor-in-Chief.

## Competing interests

The authors declare that they have no competing interests.

## Authors' contributions

Both authors interviewed and examined the patient. The draft was written by KBA, but EB contributed with references and critical review. The final draft was approved by KBA and EB.

## Pre-publication history

The pre-publication history for this paper can be accessed here:

http://www.biomedcentral.com/1471-2377/10/121/prepub
